# Frequency and predictors of individual treatment outcomes (response, remission, exacerbation, and relapse) in clinical adolescents with nonsuicidal self-injury

**DOI:** 10.1017/S0033291723001447

**Published:** 2023-12

**Authors:** Corinna Reichl, Franziska Rockstroh, Stefan Lerch, Gloria Fischer-Waldschmidt, Julian Koenig, Michael Kaess

**Affiliations:** 1 University Hospital of Child and Adolescent Psychiatry and Psychotherapy, University of Bern, Bern, Switzerland; 2 Department of Child and Adolescent Psychiatry, Centre for Psychosocial Medicine, University Hospital Heidelberg, Heidelberg, Germany; 3 Department of Child and Adolescent Psychiatry, University of Cologne, Faculty of Medicine and University Hospital Cologne, Psychosomatics and Psychotherapy, Cologne, Germany

**Keywords:** Adolescent, clinical, nonsuicidal self-injury, remission, trajectory

## Abstract

**Background:**

Nonsuicidal self-injury (NSSI) is prevalent in adolescent clinical samples. There is evidence that NSSI can be treated effectively but data on individual treatment outcomes is limited. The goal of this study was to examine response, remission, exacerbation, and relapse rates over one and two years, respectively, among a clinical sample of adolescents with NSSI. Furthermore, we aimed to identify clinically relevant predictors of NSSI trajectories.

**Methods:**

The sample consists of *n* = 203 adolescents (12–17 y., 94% female) from a specialized outpatient clinic for risk-taking and self-harming behavior with NSSI on at least five days in the six months before first assessment. Assessments were completed at baseline and one (FU1) and two (FU2) years later using structured clinical interviews and self-report questionnaires.

**Results:**

At FU1, 75% reported a reduction in NSSI frequency by at least 50% (treatment response); among those, one third (25% of the entire sample) achieved a remission (0 NSSI); an exacerbation (⩾50% more NSSI) was observed in 11% of patients. Of those in remission, 41% relapsed one year later. Predictors of non-response or non-remission were inpatient treatment and depressive symptoms. Adolescents with lower NSSI frequency at baseline had a higher risk of exacerbation. Due to limited sample size at FU2 no prediction model for relapse was established.

**Conclusions:**

While most adolescents presenting with NSSI achieved significant improvement, more attention should be paid to the rather low rates of full remission. Prediction and early detection of individuals who deteriorate during or relapse after treatment is critical.

## Introduction

Nonsuicidal self-injury (NSSI) refers to the deliberate, repetitive, and direct damage to one's own body tissue without suicidal intent (American Psychiatric Association, 2013). Emotion regulation is the most commonly reported function of NSSI (Taylor et al., 2018) and NSSI methods range from cutting to scratching, hitting, and burning. In community samples, a lifetime prevalence of 17–18% has been reported among adolescents (Muehlenkamp, Claes, Havertape, & Plener, 2012; Swannell, Martin, Page, Hasking, & John, 2014) but many individuals with NSSI seek no or delayed professional help (Lustig, Koenig, Resch, & Kaess, 2021). In adolescent clinical samples, up to 60% of patients report past NSSI (Kaess et al., 2013*a*). NSSI is commonly associated with a variety of mental disorders (Ghinea et al., 2020), adverse childhood experiences (ACE; Liu, Scopelliti, Pittman, & Zamora, 2018; McMahon, 2018), and has repeatedly been identified as the best predictor of future NSSI (Fox et al., 2015; Wichstrøm, 2009). It should be noted that in the literature, umbrella terms such as ‘direct self-injury’ or ‘self-harm’ are frequently used to describe self-injurious behaviors irrespective of their intent, hindering comparability of studies (Muehlenkamp, 2005). In the present paper, SB refers to suicidal behavior such as suicide attempts and NSSI describes nonsuicidal behavior as defined above.

Despite a lack of intent to die from the behavior, NSSI is closely linked to suicidal behavior (SB; American Psychiatric Association, 2013), and both behaviors share comorbidities and risk factors (Groschwitz et al., 2015; McMahon, 2018). The role of NSSI as a significant risk factor for SB has been established in two meta-analyses (Castellví et al., 2017; Ribeiro et al., 2016). Adolescents from a community sample with onset or maintenance of self-harm (regardless of suicidal intent) had an increased probability of SB the following year, whereas for adolescents who stopped, the risk for SB dropped to a level comparable to those who never self-harmed (Koenig et al., 2017). This confirms the directional link between self-harm and SB and highlights the potential of reducing self-harm as an important element in preventing SB. Whether the cessation of NSSI alone has similar effects was not examined.

In the general population, NSSI rates have been shown to peak during adolescence and to decline into young adulthood (Plener, Schumacher, Munz, & Groschwitz, 2015) but literature on predictors of change is scarce. Depression has been identified as a risk factor for NSSI and its maintenance over different follow-up periods (Barrocas, Giletta, Hankin, Prinstein, & Abela, 2015; Duggan, Heath, & Hu, 2015; Hankin & Abela, 2011; Plener et al., 2015). In a sample of young adults, Glenn and Klonsky (2011) examined whether cross-sectional correlates of NSSI had predictive value over one year and identified NSSI frequency and borderline personality disorder (BPD) features as prospective predictors for future NSSI. Past research reported mixed findings regarding psychiatric treatment and the longitudinal trajectory of NSSI. In a systematic review including quantitative and qualitative studies, professional help was mentioned as an important element in terminating NSSI. However, this association was only reported in qualitative interviews and was found to be less relevant than family support and intrapersonal factors (Mummé, Mildred, & Knight, 2017). In other studies, adolescents who stopped NSSI were less likely to have received treatment (Andrews, Martin, Hasking, & Page, 2013) and those who continued had a lower probability of reporting therapy as being helpful in ceasing NSSI (Whitlock, Prussien, & Pietrusza, 2015). These findings do not necessarily present an evidence for a negative treatment effect but could point to a higher general psychosocial distress in those seeking professional care. Other sources of help, particularly family, may hold more meaning for affected adolescents during the process of stopping NSSI.

Previous research on treatment outcome reported promising effects for psychotherapeutic interventions with a focus on self-harm in general, including both suicidal and nonsuicidal self-injurious behaviors (Plener et al., 2017; Turner, Austin, & Chapman, 2014). Recently, specifically developed brief interventions for NSSI, such as the Cutting Down Program (CDP; Kaess et al., 2020) and the Treatment for Self-Injurious Behaviors (T-SIB; Andover, Schatten, Morris, Holman, & Miller, 2017) showed significant reductions in NSSI frequency (Calvo et al. 2022). This, however, unfortunately does not reflect significant improvement for each patient, as individual outcomes may differ. Analogous to treatment resistant depression, there are individuals who do not respond adequately to conventional treatment (Asarnow et al., 2011; Dwyer, Stringaris, Brent, & Bloch, 2020), which can result in a smaller reduction of NSSI than anticipated or even an exacerbation over time. The examination of mean changes bears the risk of overlooking individual trajectories.

Given the diverse trajectories of most mental health problems and individual differences in treatment response, the idea of personalized treatment in psychotherapy has been discussed extensively (Cuijpers, Ebert, Acarturk, Andersson, & Cristea, 2016) but only recently is examined for any form of self-injurious behaviors (Berk et al., 2022). Due to its transdiagnostic character, NSSI may profit particularly from a personalized therapeutic approach. Identifying clinically relevant features that predict individual trajectories of NSSI is key to establish prognostic markers and inform clinical decision-making.

The aim of the present study was to examine the individual changes of NSSI frequency in a sample of help-seeking adolescents with NSSI at a specialized outpatient clinic for risk-taking and self-harming behavior. To account for individual trajectories of NSSI observed in clinical populations, we examined subgroups according to reported one-year change following certain criteria: We differentiated between a *response* if NSSI frequency was reduced by at least 50%, a *remission* with a complete cessation of NSSI after one year, and an *exacerbation* in NSSI frequency with a twofold increase of NSSI. Additionally, we explored potential *relapses* among patients showing remission another year later. Clinically relevant predictors of group membership were identified to study potential markers for the early distinction of patients experiencing an improvement or aggravation in the following year, and to generate potential targets for personalized medicine in the treatment of adolescent NSSI.

## Methods

### Participants and procedure

The sample consists of adolescents (12–17 years) who presented at the specialized outpatient clinic for adolescent risk-taking and self-harming behavior at the Department of Child and Adolescent Psychiatry, University Hospital Heidelberg, Germany (AtR!Sk; Ambulanz für Risikoverhalten und Selbstschädigung). Please refer to Kaess et al. (2020, 2017) for more details regarding the specialized outpatient clinic AtR!Sk.

Patients were recruited consecutively and included in the AtR!Sk cohort study after signing written informed consent. For participants under the age of 16, the parents’ written consent was obtained. The local ethics committee (ID S-449/2013) approved the AtR!Sk cohort study and compliance with the Declaration of Helsinki (World Medical Association, 2013) was ensured. The present analyses only include individuals who reported repetitive engagement in NSSI (at least on five days in the previous six months) at the time of baseline assessment.

### Assessments

Specially trained clinicians conducted structured clinical assessments at baseline and after one (FU1) and two (FU2) years, respectively. The following interview- and questionnaire-based assessments were conducted at baseline as well as at FU1 and FU2. Each assessment instrument was conducted in the respective validated German version.

The Self-Injurious Thoughts and Behaviors Interview (SITBI-G; Fischer et al., 2014) was applied to examine *NSSI* frequency and methods. The 6-month frequency of NSSI at baseline (‘How many times in the past six months have you purposely hurt yourself without wanting to die?’) was included as a predictor variable in analyses and the outcome grouping variables were created using the difference from baseline to follow-up data from this item (see statistical analyses for further details). In accordance with the definition of NSSI of the Diagnostic and Statistical Manual of Mental Disorders (DSM-5; American Psychiatric Association, 2013), we rated NSSI events per day resulting in a maximum of 183 NSSI behaviors during a time period of six months. The SITBI-G has good psychometric properties (Fischer et al., 2014). *Borderline personality disorder* (*BPD*) was measured using the BPD module of the Structured Clinical Interview for DSM-IV Axis II (SCID-II; Wittchen, Zaudig, & Fydrich, 1997). On a scale from 1 (not fulfilled), 2 (partially fulfilled) to 3 (fulfilled), all nine BPD-criteria were rated. As an indicator of severity, the number of criteria rated as 3 were added up. Previous analyses by our research group revealed excellent interrater reliability (Cohen's ĸ = 1.00) for this interview (Kaess et al., 2013*b*). Mental disorders were assessed using the structured Mini-International Neuropsychiatric Interview for Children and Adolescents (M.I.N.I.-KID; Sheehan et al., 2010). Finally, assessors rated the Clinical Global Impression Scale (CGI-S; Busner & Targum, 2007) at the end of the diagnostics appointment as an indicator of *general symptom severity*. Demographic data, such as *age, sex, school type* and *living situation* were assessed using standardized interview questions. At follow-up, participants reported the usage of any form of treatment and medication they had received in the past year. Dose of treatment (outpatient treatment sessions; days of inpatient treatment) was also assessed.

*Depression* severity was assessed using the Depression Inventory for Children and Adolescents (DIKJ; Stiensmeier-Pelster, Schürmann, & Duda, 1991). Twenty-seven items covering all substantial DSM-IV criteria were rated on a scale from 0 (no symptomatology) to 2 (high severity). We found good internal consistency (Cronbach's *α* = 0.88) in the present study. To assess *adverse childhood experiences* (*ACE*), the Childhood Experience of Care and Abuse Questionnaire (CECA.Q; Kaess et al., 2011) was conducted. This questionnaire measures antipathy, neglect, and physical and sexual abuse by the mother and/or the father or alternative parental figures. Number of ACE by any parent were counted and summed to a value between 0 (no ACE) and 4 (all forms of ACE) in accordance with a dose-response effect (Bifulco, Bernazzani, Moran, & Jacobs, 2005). For the German translation, Kaess et al. (Kaess et al. 2011) reported good to excellent psychometric properties across different types of ACE.

### Statistical analysis

Participants who attended the 12-months follow-up (FU1) and reported NSSI on at least five days during the six months before baseline were included in the present analyses. To account for the fact that NSSI often does not cease immediately after seeking treatment, 6-month time periods relative to respective assessments were considered when examining changes in NSSI frequency: The frequency of NSSI in the six months before baseline was compared to six months before FU1.

**Figure 1.**
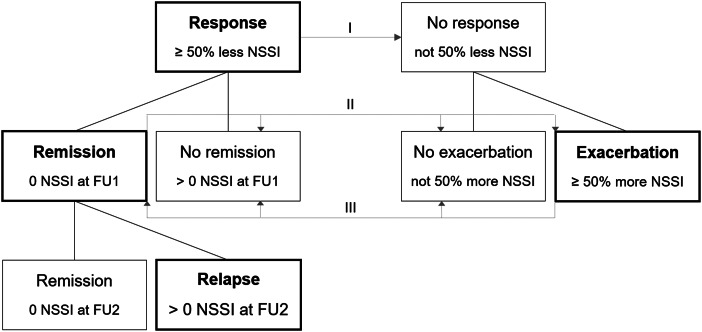
Groups according to change in non-suicidal self-injury frequency from baseline to follow-up. *Note.* NSSI, non-suicidal self-injury; FU, follow-up. ^I^ Model 1: Response v. No Response. ^II^ Model 2: Remission v. No remission, no exacerbation, exacerbation. ^III^ Model 3: Exacerbation v. Remission, no remission, no exacerbation.

Participants were classified within groups according to their individual change in NSSI frequency from baseline to FU1: As presented in Figure 1, we differentiated between response and non-response in a first step. *Response* was defined as a reduction of NSSI frequency of at least 50% of days within a time interval of six months one year later. *Non-responders*, on the other hand, did not show a reduction of NSSI frequency of at least 50%. In a second step, both groups were further divided into subgroups. If participants with a response did not report any incidents of NSSI within six months prior to the follow-up assessment, they were assigned to the *remission* group and adolescents in the *non-remission* group had a reduction but no full remission. Within non-responders, a distinction was made between adolescents in the *exacerbation* group who reported an increase of NSSI frequency of at least 50% and those who neither improved not deteriorated (*non-exacerbation*). Among patients with a remission at FU1, the *relapse* rates at FU2 were examined if data were available. For better comprehensibility and clarity, the present analyses focused on response, remission, exacerbation, and relapse as the clinically most relevant groups.

Sample characteristics were calculated using descriptive statistics. For testing the significance of change in NSSI frequency, the Wilcoxon signed-rank test was used and effect size was calculated according to Fritz et al. (2012). Logistic regression analyses were computed with group membership as the respective dichotomous outcome variable (e.g. *response* no/yes). Due to a limited sample size at FU2, relapses were only reported as descriptive statistics and no logistic regression model was established. To ensure comparability, predictor variable values were standardized. Analyses were performed using Stata/SE (Version 16.0, Stata Corp LLC, College Station, TX, USA) and the alpha-level was set to 0.05.

## Results

### Descriptive statistics

Out of *n* = 625 adolescent outpatients participating in the AtR!Sk cohort study (participation rate 86%), *n* = 428 fulfilled inclusion criteria of NSSI on at least five days in the six months before baseline, and *n* = 240 provided FU1 data (follow-up rate 56%). Due to missing questionnaire data, *n* = 37 participants were excluded from analyses, resulting in a sample of *n* = 203. Drop-out analyses are presented as online Supplementary material. Sociodemographic and clinical sample characteristics are presented in Table 1.

**Table 1.** Sample characteristics by group

	Total *n* = 203	Response *n* = 152	Remission *n* = 51	Exacerbation *n* = 23	Relapse *n* = 11
Age, *M* (s.d.)	14.89 (1.45)	14.89 (1.46)	14.67 (1.52)	14.96 (1.33)	15.09 (1.38)
Female sex, *n* (*%*)	190 (93.6)	142 (93.4)	46 (90.2)	21 (91.3)	10 (90.9)
School, *n* (*%*)^a^					
Gymnasium	89 (43.8)	63 (41.5)	16 (31.4)	11 (47.8)	6 (54.6)
Realschule	65 (32.0)	52 (34.2)	23 (45.1)	8 (34.8)	3 (27.3)
Hauptschule	19 (9.4)	16 (10.5)	7 (13.7)	2 (8.7)	1 (9.1)
Other	30 (14.8)	21 (13.8)	5 (9.8)	2 (8.7)	1 (9.1)
Living situation, *n* (*%*)					
Both parents	95 (47.5)	72 (48.3)	21 (41.2)	12 (52.2)	4 (36.4)
One parent	74 (37.0)	52 (34.9)	12 (37.3)	5 (21.7)	6 (54.6)
Other living situation (e.g. youth welfare)	31 (15.5)	25 (16.8)	11 (21.6)	6 (26.1)	1 (9.1)
Treatment, *n* (*%*)					
Outpatient	170 (83.7)	123 (80.9)	36 (70.6)	20 (87.0)	8 (72.7)
Inpatient	89 (43.8)	55 (36.2)	7 (13.7)	13 (56.5)	2 (18.2)
Psychotropic medication, *n* (%)	50 (24.6)	32 (21.05)	6 (11.8)	4 (17.4)	3 (27.3)
Diagnoses, *n* (*%*)^b^					
F1 Mental and behavioral disorders due to psychoactive substance use	36 (17.7)	31 (20.4)	14 (27.5)	3 (13.0)	6 (54.5)
F3 Affective disorders	155 (76.4)	113 (74.3)	35 (68.6)	18 (78.3)	8 (72.7)
F4 Neurotic, stress-related and somatoform disorders	86 (42.4)	62 (40.8)	20 (39.2)	10 (43.5)	5 (45.5)
F5 Behavioral syndromes associated with physiological disturbances and physical factors	31 (15.3)	23 (15.1)	5 (9.8)	3 (13.0)	0 (0.0)
F8 Disorders of psychological development	1 (0.5)	1 (0.7)	1 (2.0)	0 (0.0)	0 (0.0)
F9 Behavioral and emotional disorders with onset usually occurring in child and adolescence	36 (17.7)	31 (20.4)	17 (33.3)	2 (8.7)	3 (27.3)
NSSI frequency baseline, *M* (s.d.)	52.19 (45.64)	53.8 (47.13)	46.1 (43.48)	22.70 (17.05)	59.18 (57.90)
NSSI frequency follow-up, *M* (s.d.)	19.28 (31.01)	6.28 (10.59)	0.00 (0.00)	53.48 (34.50)	7.27 (7.16)^c^
Depression, *M* (s.d.)	32.18 (8.71)	32.00 (9.31)	29.53 (10.26)	30.96 (7.71)	28.82 (9.64)
BPD, *M* (s.d.)	3.81 (2.06)	3.81 (2.07)	3.67 (2.18)	3.65 (2.19)	3.64 (2.80)
ACE score, *M* (s.d.)	1.48 (1.25)	1.47 (1.28)	1.33 (1.31)	1.91 (1.28)	1.09 (1.38)
General symptom severity, *M* (s.d.)	5.11 (0.88)	5.16 (0.88)	5.06 (1.07)	4.96 (0.98)	5.09 (0.83)

*Note*. *M*, mean; *SD*, standard deviation; *n*, sample size; NSSI, nonsuicidal self-injury; BPD, borderline personality disorder; ACE, adverse childhood experiences.

a German educational categories include Gymnasium = secondary school terminating with a general qualification for university, Realschule = secondary school terminating with a secondary school level-I certificate, Hauptschule = secondary elementary school.

b F0 (organic mental disorders), F2 (schizophrenia, schizotypal and delusional disorders), F7 (mental retardation), were not fulfilled by any patient. Frequency of F6 (disorders of adult personality and behavior) is not reported since only the SCID-II BPD module was conducted.

c For relapses, 12-months frequency at follow-up 2 is reported.

The most commonly reported methods of NSSI were cutting or carving (99%), scraping the skin (47%), manipulating a wound (45%), and hitting oneself (37%). On average, adolescents reported the use of three different NSSI methods (*M* = 3.37, s.d. = 2.05). The frequency of NSSI in the six months before baseline ranged between five and 180 with a mean of 52.19 (s.d. = 45.64). One year later, this number decreased significantly to 19.28 days with NSSI (s.d. = 31.01) over the same period of 6 months in the full sample (*z* = 8.93, *p* < 0.001, *r* = 0.63).

The majority of participants received outpatient therapy between baseline and FU1 (*n* = 170) and reported a mean number of 24.29 sessions (s.d. = 18.79). If any inpatient treatment was provided (*n* = 89), this lasted 71.39 days (s.d. = 63.64) on average. Of outpatients, 48% were treated at the specialized outpatient clinic AtR!Sk with either the CDP (10 single sessions; 36%), dialectical behavior therapy for adolescents (DBT-A; 25 single sessions and 20 sessions of skills training; 53%), or both (11%). Other outpatient treatment options that were provided outside AtR!Sk consisted of cognitive behavioral therapy (35%), psychodynamic methods (5%) or others (13%). In addition to standard inpatient care (63%), stays in acute inpatient units (51%) and day clinics (20%) were reported by those receiving inpatient treatment between baseline and FU1. Many patients reported a combination of different treatment types. At FU1, *n* = 50 (25%) reported taking at least one form of any psychotropic medication in the past year. The most commonly named form of medication was antidepressants (*n* = 44, 88%), followed by neuroleptics (*n* = 11, 22%).

### Individual outcomes

**Figure 2.**
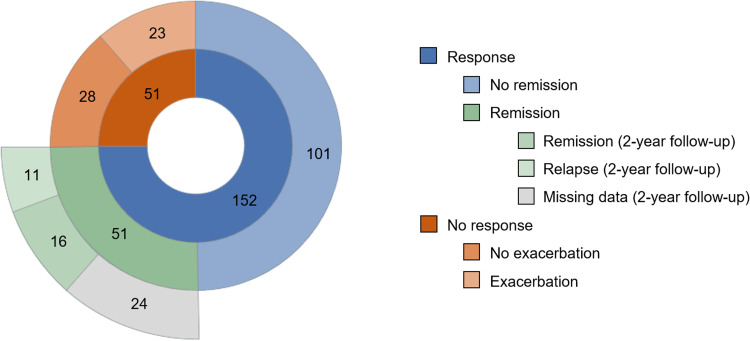
Distribution and group sizes.

In the response group (*n* = 152; 75%), NSSI frequency dropped from *M* = 53.79 (s.d. = 47.13) to *M* = 6.28 (s.d. = 10.59) and among non-responders (*n* = 51; 25%), NSSI increased from *M* = 47.41 (s.d. = 40.97) to *M* = 58.02 (s.d. = 38.74) over one year. Per definition, there were zero incidents of NSSI in the remission group (*n* = 51; 25%) at FU1. The exacerbation group (*n* = 23; 11%) reported NSSI on *M* = 22.70 (s.d. = 17.05) days at baseline and *M* = 53.48 (s.d. = 34.50) at FU1. Among participants with neither a response nor an exacerbation (*n* = 28; 14%), NSSI frequency was *M* = 67.71 (s.d. = 43.86) at baseline and *M* = 61.75 (s.d. = 42.16) after one year. Figure 2 illustrates the distribution and sample size of groups according to change.

Out of *n* = 51 participants with a remission at FU1, *n* = 27 provided FU2 data. Out of those, *n* = 11 (41%) reported to have relapsed and self-injured at some point between FU1 and FU2. Four participants only relapsed once, and remaining adolescents reported between four and twenty incidents of NSSI.

### Logistic regression models

Table 2 presents logistic regression analyses for all three previously defined models. Among univariate models, the duration of inpatient treatment (*OR* = 0.56, *p* < 0.001) and medication use (*OR* = 0.49, *p* = 0.043) were significant negative predictors of response. In multivariate analyses, inpatient treatment remained significant (*OR* = 0.48, *p* < 0.001) and general symptom severity also reached significance (*OR* = 1.81, *p* = 0.006). No or shorter inpatient stays and higher general symptom severity at baseline were therefore linked to a higher probability of a response. When controlling for inpatient treatment, medication lost significance as a predictor (*OR* = 0.88, *p* = 0.784).

**Table 2.** Univariate and multivariate logistic regression models for response, remission, and exacerbation

	univariate				multivariate			
	*OR*	*CI*	*p*	*R2*	*OR*	*CI*	*p*	*R2*
Model I: Response – No Response								
Age	1.00	0.73; 1.38	0.980	0.00	1.11	0.75; 1.64	0.591	
Sex^a^	1.13	0.30; 4.26	0.861	0.00	0.69	0.16; 2.89	0.609	
Outpatient treatment	0.91	0.66; 1.24	0.533	0.00	0.89	0.63; 1.24	0.490	
Inpatient treatment	0.56	0.41; 0.76	< 0.001	0.10	0.48	0.32; 0.71	< 0.001	
Psychotropic medication^a^	0.49	0.24; 0.98	0.043	0.03	0.88	0.35; 2.23	0.784	
NSSI frequency	1.16	0.83; 1.62	0.388	0.01	1.25	0.84; 1.87	0.266	
Depression	0.92	0.67; 1.27	0.606	0.00	0.93	0.63; 1.37	0.711	
BPD	1.00	0.73; 1.38	0.987	0.00	0.78	0.51; 1.18	0.237	
ACE score	0.97	0.71; 1.33	0.858	0.00	0.88	0.59; 1.39	0.525	
General symptom severity	1.25	0.91; 1.70	0.171	0.01	1.81	1.18; 2.77	0.006	0.17
Model II: Remission – No Remission								
Age	0.82	0.59; 1.12	0.211	0.01	0.81	0.55; 1.19	0.283	
Sex^1^	1.96	0.61; 6.28	0.259	0.01	1.89	0.50; 7.11	0.345	
Outpatient treatment	0.76	0.53; 1.08	0.122	0.02	0.79	0.53; 1.20	0.269	
Inpatient treatment	0.28	0.13; 0.62	0.002	0.14	0.30	0.13; 0.70	0.006	
Psychotropic medication^a^	0.33	0.13; 0.82	0.017	0.05	0.78	0.26; 2.33	0.660	
NSSI frequency	0.83	0.59; 1.16	0.271	0.01	0.81	0.56; 1.18	0.278	
Depression	0.67	0.49; 0.92	0.013	0.04	0.68	0.47; 0.99	0.045	
BPD	0.91	0.66; 1.26	0.571	0.00	1.05	0.69; 1.60	0.808	
ACE score	0.85	0.61; 1.18	0.325	0.01	0.90	0.61; 1.32	0.582	
General symptom severity	0.93	0.68; 1.27	0.643	0.00	1.29	0.86; 1.95	0.217	0.21
Model III: Exacerbation – No Exacerbation								
Age	1.06	0.68; 1.64	0.806	0.00	0.97	0.57; 1.63	0.896	
Sex^1^	1.46	0.30; 7.06	0.635	0.00	3.78	0.59; 24.34	0.162	
Outpatient treatment	1.19	0.80; 1.79	0.391	0.01	1.35	0.86; 2.12	0.187	
Inpatient treatment	1.08	0.71; 1.62	0.729	0.00	1.74	0.96; 3.18	0.069	
Psychotropic medication^a^	0.61	0.20; 1.90	0.396	0.01	0.29	0.06; 1.35	0.115	
NSSI frequency	0.25	0.10; 0.62	0.003	0.15	0.22	0.08; 0.59	0.002	
Depression	0.86	0.56; 1.31	0.473	0.01	1.06	0.63; 1.80	0.827	
BPD	0.92	0.59; 1.42	0.700	0.00	1.02	0.59; 1.77	0.937	
ACE score	1.46	0.95; 2.24	0.083	0.03	1.70	1.00; 2.90	0.050	
General symptom severity	0.83	0.55; 1.26	0.381	0.01	0.66	0.37; 1.17	0.154	0.23

*Note. OR*, odds ratio; *CI*, 95% confidence interval; *p*, p-value; *R2*, Nagelkerke Pseudo R2; NSSI, nonsuicidal self-injury; BPD, borderline personality disorder; ACE, adverse childhood experiences.

a Unstandardized.

Longer inpatient treatment was also found to negatively predict remission compared to non-remission in both uni- and multivariate models (*OR* = 0.28, *p* = 0.002; *OR* = 0.30, *p* = 0.006). Again, medication intake negatively predicted remission (*OR* = 0.33, *p* = 0.017) but not when including covariates (*OR* = 0.78, *p* = 0.660). Furthermore, depression was identified as a significant negative predictor in the uni- and multivariate models for remission (*OR* = 0.67, *p* = 0.013; *OR* = 0.68, *p* = 0.045). Higher depression severity at baseline was associated with a decreased probability of a remission one year later.

Exacerbation was predicted by 6-month NSSI frequency in both models (*OR* = 0.25, *p* = 0.003; *OR* = 0.22, *p* = 0.002): less NSSI at baseline increased the probability for exacerbation one year later.

## Discussion

This study focused on the investigation of individual treatment outcomes of NSSI among treatment-seeking adolescents. In a first step, we analyzed the frequencies of response, remission, exacerbation, and relapse of NSSI in this high-risk sample. Some results were overall encouraging: Three quarters were responders and reduced NSSI frequency at least by half, and – with almost 90% less NSSI events after one year – the response group without full remission displayed a vast improvement. However, and as commonly not reflected by mean symptom reductions, only one quarter of patients reported full remission of NSSI despite in many cases receiving evidence-based mental healthcare. Furthermore, one in ten patients showed an exacerbation of NSSI frequency one year later. Finally, out of patients with a remission after one year, another year later around two fifths relapsed, though in many cases relapse referred to NSSI on only one day. Considering the sample composition with high levels of psychopathology and low psychosocial functioning, the high response rates are notable and encouraging. However, our results show considerable heterogeneity in individual trajectories within a specialized outpatient service for adolescents with NSSI, that can clearly deviate from the overall positive mean outcomes.

Our findings are in line with studies on depression and self-harm reduction in adolescence. Treatment-resistant depression is common and between 30–40% of adolescent patients do not respond adequately to evidence-based first line treatment (Dwyer et al., 2020). Patients with treatment-resistant depression often report NSSI and SB and such behaviors can persist aligned with depressive symptomatology (Asarnow et al., 2011). The response rate in the present study is comparable to recently published data on self-harm trajectories. Using latent class analysis, Berk et al. (2022) reported improvement of NSSI and SB in 74% of patients receiving either DBT or individual and group supportive therapy over 6 to 12 months. Interestingly, in their analyses non-response was predicted by externalizing symptoms rather than internalizing, such as depression, which should be considered as an often overlooked but possibly crucial factor hindering self-harm treatment (Witte, Gauthier, Huang, Ribeiro, & Franklin, 2018). The slightly higher response rates of NSSI compared to depression may in part be attributed to the nature of both phenomena and their treatment. NSSI is a definable and often observable behavior which can be targeted by skills training and may respond rather quick to intervention. During treatment, the reduction of self-injury is often a first step in a longer process of improving emotion regulation and profound dysfunctional assumptions. The sustainability of NSSI reduction may depend on the long-term changes in underlying thought patterns. Further research into NSSI treatment response and resistance may promote intervention tailoring and advance development of personalized treatment for related disorders, such as depression.

Cessation of NSSI was only reported by one in four adolescents and many had relapsed another year later. This raises the question whether the commonly used definition of remission as a complete cessation adequately depicts the true course of NSSI. Lewis, Kenny, Whitfield, and Gomez (2019) found that while complete cessation was an essential part of recovery, patients often reported it to be one piece in a bigger process. Even after having stopped NSSI, sometimes for years, many participants did not consider themselves to be recovered as long as thoughts and urges remained which was reinforced by the possibility of relapses. More analyses are needed regarding NSSI remission and relapse to gain a realistic concept of what lasting NSSI recovery may look like and how it can be achieved. Furthermore, the topic of biological underpinnings of NSSI should briefly be addressed. Researchers have made progress in recent years in identifying neurobiological states, correlates, and predictors of NSSI such as e.g. immunological markers, altered HPA functioning, and pain sensitivity (Kaess et al., 2021). However, little is known about temporal mechanisms linking biomarkers to NSSI and about the effect biological systems may have on the persistence of NSSI, which should be examined in future studies.

Some adolescents did not improve and one in ten even deteriorated between first contact and one-year follow-up. This negative direction of the trajectory has not been examined in previous literature and this small but potentially highly burdened group has been neglected in research so far. Particularly, the association between NSSI exacerbation with psychopathology and psychosocial functioning should be examined closely and to prevent increases in NSSI frequency and detect changes during treatment, early warning signs need to be identified.

The second goal was to identify clinically relevant predictors of NSSI outcome one year after first presentation at the outpatient clinic. Results varied between groups: Adolescents who received longer inpatient treatment after their baseline assessment had a significantly lower probability of attaining a response or remission. This finding is in line with community-based studies (Andrews et al., 2013; Whitlock et al., 2015) and has been similarly shown in inpatient settings (Ougrin et al., 2021). Different interpretations are possible. On the one hand, adequate care is often sought out by individuals with severe mental health problems (Zachrisson, Rödje, & Mykletun, 2006) and the initiation of inpatient treatment speaks for particularly high levels of psychopathology and poor psychosocial functioning. A non-response in inpatients may be a sign of general psychosocial stress beyond and not limited to NSSI that may not have been captured by the baseline assessment that was adjusted for. The need for inpatient treatment may not have been apparent at baseline but was a result of an escalation of impairment over time. On the other hand, an inpatient unit may not be the appropriate environment for treating NSSI. A psychiatric hospitalization can be a stressor in itself and patients are, in addition to their own burden, confronted with others’ distress and self-harm (Haynes, Eivors, & Crossley, 2011; James, Stewart, & Bowers, 2012; Timberlake, Beeber, & Hubbard, 2020). This can lead to difficult group dynamics which may be met with NSSI as a coping strategy. Our findings can be interpreted as support for NSSI treatment guidelines by The Association of the Scientific Medical Societies in Germany (Plener et al., 2017), that generally recommend giving priority to out- over inpatient treatment under the prerequisite of safety measures. Further, our findings of inpatient treatment being negatively related to a decrease in NSSI frequency may in part be explainable by the fact that a substantial part of adolescents received outpatient treatment at the outpatient unit AtR!Sk. The AtR!Sk therapy program is specialized in the treatment of self-harming and risk-taking behaviors and (sub)syndromal BPD. Adolescents receive treatment according to a brief cognitive-behavioral psychotherapy manual (CDP), and alternatively or additionally DBT-A, both of which have been shown to be efficient and effective in the reduction of mean NSSI frequency (Kaess et al., 2020; Mehlum et al., 2019, 2014). Overall, our results suggest outpatient programs may be more effective in the reduction of NSSI than inpatient treatments, which are often not specialized in the treatment of self-harming behavior and may even, as discussed above, have iatrogenic effects.

In addition, when controlling for inpatient treatment and other covariates, general symptom severity also reached significance in the prediction of response. Higher levels of general symptom severity at baseline were positively linked to response, which may be explained by a greater potential for improvement in those with initially higher levels of psychopathology and lower levels of psychosocial functioning who, at the same time, were able to receive adequate care in an outpatient setting. Psychotropic medication, on the other hand, negatively predicted a response as a univariate variable but lost significance when adding other covariates to the model. This finding can be explained analog to the effect of inpatient treatment. Individually, medication intake is generally associated with higher psychopathology which reduces the probability of a response (or remission) of NSSI. When controlling for severity by including inpatient treatment, however, this effect seems to be covered and medication no longer has any predictive value.

In line with previous studies, depression was identified as a negative predictor of remission (Barrocas et al., 2015; Duggan et al., 2015; Hankin & Abela, 2011; Plener et al., 2015). Patients with more severe levels of depression at baseline were significantly less likely to achieve a NSSI remission in the following year, however, depression had no significant effect on response without complete remission. This finding may seem surprising after greater general symptom severity was found to positively predict response, as discussed in the last paragraph. It should be noted, however, that the outcome was not the same. The response group seems to be distinct from the remission group in this regard which may in part be explained by the respective definition of the groups: A response describes a significant reduction of NSSI and therefore contains the baseline value of NSSI. In line with the concept of a regression towards the mean, a higher base level of NSSI (and general symptom severity) allows for a sharper decrease and therefore an increased likelihood of a response. Remission, however, is independent of the initial NSSI rates and can be reported irrespective of past NSSI frequency. This difference could explain why higher general symptom severity predicted a response but not a remission. Furthermore, depression is a specific psychiatric disorder and general symptom severity, as measured in this study, not only includes degree of symptomatology but also psychosocial functioning. This was rated by clinicians whereas depression was measured using a self-rating questionnaire. As discussed in the limitations, questionnaires are often used for screening purposes and may overestimate severity of depressive symptoms. Interestingly and in contrast to Glenn and Klonsky (2011), severity of BPD had no influence on the likelihood of a remission and independent of its persistent character we found no indication of elevated BPD symptomatology resulting in less favorable outcomes concerning NSSI.

Finally, exacerbation was predicted by lower NSSI rates at baseline. Adolescents with less NSSI therefore had a higher probability of reporting an increase after 12 months. This could be expected since lower baseline-rates may double more quickly compared to high frequency behaviors. We also found the FU1 rates of NSSI in the exacerbation group to be comparable to baseline rates in the response group. There seemed to be a temporal shift in NSSI frequency between groups and adolescents experiencing an exacerbation may reach a peak in NSSI later, further illustrating variance in the timing of first clinical presentation. Due to the small group size no statement can be made regarding the trajectory of NSSI at FU2 and whether the frequency increased further or decreased analogous to the response group. Unfortunately, research on the rise of NSSI over time on an individual level is scarce and adolescents showing an aggravation in NSSI symptoms may require particular attention. Lastly and in addition to BPD, neither age, sex, nor dose of outpatient treatment had any predictive value in the prediction of change in NSSI frequency.

Some limitations should be noted. The sex ratio was clearly unbalanced with most of the sample being female. Although an effect could not be detected in the present data it cannot be ruled out that the longitudinal trajectory of NSSI differs depending on sex. As presented in the supplement (see online Supplementary Table S1), there was a small but significant effect of sex and NSSI frequency on drop-out. Male patients had higher drop-out rates as well as adolescents with lower NSSI rates at baseline, which may be explainable by lower feelings of identification with the study's target group. Due to drop-out, this cannot be confirmed. Furthermore, depression severity and ACE scores were assessed using questionnaires, which are mainly used for screening purposes. This may lead to an overestimation of symptoms compared to extensive clinical interviews we conducted to assess NSSI and BPD. Also, due to small sample sizes at FU2 no prediction model could be established for relapse. This is the first study using a more individualistic approach into examining trajectories of NSSI frequency by defining corresponding groups instead of mean changes. By identifying predictors of response, remission, and exacerbation, this study added to the important discussion of personalized treatment options in mental health care. Additionally, the sample size and composition of help-seeking adolescents as well as the longitudinal study design should be noted.

## Conclusion

In line with previous research, we found high levels of response over one year in this high-risk adolescent sample. Complete remission, though, was rare and a small but considerable group reported an exacerbation of NSSI frequency. This highlights the heterogeneity of NSSI treatment outcomes and the importance of accounting for individual processes in the study of self-harming behaviors. Inpatient treatment and depression severity were identified as clinically relevant factors that may hinder a response or remission whereas general symptom severity increased the likelihood for a response. The negative effect of inpatient care on NSSI frequency endorses the general recommendation of favoring out- over inpatient settings for treating NSSI and should be taken into consideration in the clinical decision-making process. Furthermore, lower NSSI frequency at baseline is not necessarily an all-clear signal since it was found to elevate the risk of exacerbation. Early detection of patients with an increase in NSSI after seeking help is critical and further research in the development and promotion of personalized treatment options is clearly indicated.

## Supporting information

Reichl et al. supplementary material Reichl et al. supplementary material

## Supplementary material

The supplementary material for this article can be found at https://doi.org/10.1017/S0033291723001447.
