# Supplementary material for: Frequency and predictors of individual treatment outcomes (response, remission, exacerbation, and relapse) in clinical adolescents with nonsuicidal self-injury
Source: Psychol Med. 2023 Jun 7;53(16):7636–45. doi: 10.1017/S0033291723001447 (PMC10755228; doi:10.1017/S0033291723001447)
Supplement: Reichl et al. supplementary material [file S0033291723001447sup001.docx]

**Supplementary material**

**Baseline characteristics – drop-out analyses**

To determine baseline differences between participants who dropped out (*n* = 225) or returned for follow-up assessments and provided complete data (*n* = 203), supplementary analyses were conducted. With completeness as the dichotomous outcome, logistic regression analyses were computed for all sociodemographic and clinical variables that were collected at baseline and included in main analyses. Grouping variables (response, remission, exacerbation, relapse) and information regarding psychotherapy could not be included since relevant data was only assessed at FU.

As presented in Table S1, sex and NSSI frequency at baseline were significant predictors of data completeness. Male adolescents were less likely to return for FU1 (*OR* = 0.46, *p* = .027; *OR* = 0.42, *p* = .021) while adolescents with higher rates of NSSI at baseline had a higher probability of completing FU1 assessments (*OR* = 1.24, *p* = .031; *OR* = 1.25, *p* = 0.55).

The implications of these results are discussed in the main manuscript.

|  | univariate | | | |  |  | multivariate | | | |
| --- | --- | --- | --- | --- | --- | --- | --- | --- | --- | --- |
|  | *OR* | *CI* | *p* | *R2* |  |  | *OR* | *CI* | *p* | *R2* |
| **Completeness** | | | | | | | | | | |
| Age | 1.00 | 0.83; 1.21 | 0.987 | 0.00 |  |  | 1.18 | 0.94; 1.49 | 0.148 |  |
| Sex^a^ | 0.46 | 0.23; 0.92 | 0.027 | 0.02 |  |  | 0.42 | 0.20; 0.87 | 0.021 |  |
| NSSI frequency | 1.24 | 1.02; 1.50 | 0.031 | 0.02 |  |  | 1.25 | 1.00; 1.56 | 0.055 |  |
| Depression | 1.11 | 0.91; 1.35 | 0.315 | 0.00 |  |  | 1.14 | 0.91; 1.44 | 0.256 |  |
| BPD | 0.87 | 0.72; 1.06 | 0.169 | 0.01 |  |  | 0.85 | 0.66; 1.08 | 0.187 |  |
| ACE score | 1.02 | 0.84; 1.24 | 0.861 | 0.00 |  |  | 0.97 | 0.77; 1.21 | 0.777 |  |
| General symptom severity | 0.95 | 0.78; 1.15 | 0.575 | 0.00 |  |  | 0.91 | 0.72; 1.16 | 0.464 | 0.05 |

Table S1

*Univariate and multivariate logistic regression models for data completeness*

*Note.* *OR* = odds ratio, *CI* = 95% confidence interval, *p* = p-value, *R2* = Nagelkerke Pseudo R2, NSSI = nonsuicidal self-injury, BPD = borderline personality disorder, ACE = adverse childhood experiences.
^a^ Sex is unstandardized.
